# Bactericidal Properties of Plants-Derived Metal and Metal Oxide Nanoparticles (NPs)

**DOI:** 10.3390/molecules23061366

**Published:** 2018-06-06

**Authors:** Sin-Yeang Teow, Magdelyn Mei-Theng Wong, Hooi-Yeen Yap, Suat-Cheng Peh, Kamyar Shameli

**Affiliations:** 1 Department of Medical Sciences, School of Healthcare and Medical Sciences, Sunway University, Jalan Universiti, Bandar Sunway, Subang Jaya 47500, Selangor Darul Ehsan, Malaysia; magdelyn.w@gmail.com (M.M.-T.W.); 17084351@imail.sunway.edu.my (H.-Y.Y.); pehsc@sunway.edu.my (S.-C.P.); 2 Anatomical Pathology Department, Sunway Medical Centre, Jalan Lagoon Selatan, Bandar Sunway, Subang Jaya 47500, Selangor Darul Ehsan, Malaysia; 3 Department of Environment and Green Technology, Malaysia-Japan International Institute of Technology, Universiti Teknologi Malaysia, Jalan Sultan Yahya Petra, Kuala Lumpur 54100, Malaysia; kamyar@utm.my

**Keywords:** antibacterial, nanoparticles, green synthesis, biomedical applications, plants

## Abstract

Nanoparticles (NPs) are nano-sized particles (generally 1–100 nm) that can be synthesized through various methods. The wide range of physicochemical characteristics of NPs permit them to have diverse biological functions. These particles are versatile and can be adopted into various applications, particularly in biomedical field. In the past five years, NPs’ roles in biomedical applications have drawn considerable attentions, and novel NPs with improved functions and reduced toxicity are continuously increasing. Extensive studies have been carried out in evaluating antibacterial potentials of NPs. The promising antibacterial effects exhibited by NPs highlight the potential of developing them into future generation of antimicrobial agents. There are various methods to synthesize NPs, and each of the method has significant implication on the biological action of NPs. Among all synthetic methods, green technology is the least toxic biological route, which is particularly suitable for biomedical applications. This mini-review provides current update on the antibacterial effects of NPs synthesized by green technology using plants. Underlying challenges in developing NPs into future antibacterials in clinics are also discussed at the present review.

## 1. Nanoparticles and Green Technology

**Figure 1:**
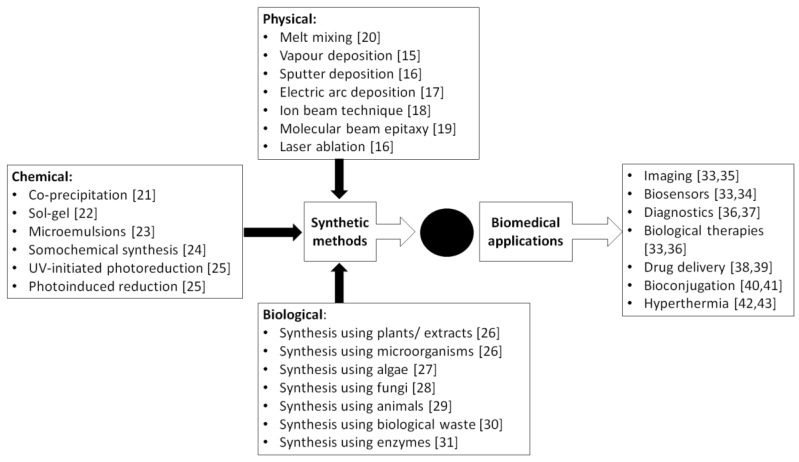
Current synthetic methods of nanoparticles (NPs) and their biomedical applications. The core methods used for NPs construction are divided into physical, chemical, and biological methods. The generated NPs can be utilized in various biomedical applications including imaging, biosensors, diagnostics, biological therapies, drug delivery, bioconjugation, and hyperthermia.

Nanoparticles (NPs), also known as nanomaterials, are small on a molecular scale and have various physical and chemical properties. Some examples of NPs are made up of metals ions such as Au, Ag, Pd, Pt, Zn, Fe, and Cu, and metal oxides such as Ag_2_O, NiO, ZnO, CuO, FeO, and CeO_2_. The advancement of nanotechnology has also given rise to the development of various nanocomposites, which are multiphase solid materials consisting of multiple types of NPs and polymers to improve single-metal biological effects and overcome structure-function related issues [1]. Multiple biological actions of the NPs such as antibacterial [2,3], antioxidant [2,4], anticancer [5,6], antifungal [5,7], antiviral [8,9], antiparasitic [10,11] and anti-inflammatory activities [12,13] have been associated with their highly diverse chemistry-rich characteristics [14]. There are myriad ways of synthesizing NPs, including physical (e.g., vapor deposition [15], sputter deposition [16], electric arc deposition [17], ion beam technique [18], molecular beam epitaxy [19], melt mixing [20]), chemical (e.g., co-precipitation [21], sol-gel [22], microemulsions [23], sonochemical synthesis [24] UV-initiated photoreduction [25]), and biological (e.g., synthesis using plant extracts [26], microorganisms [26], algae [27], fungi [28], animals [29] or agricultural waste [30], enzymes [31]) methods as well as hybrid methods [32] (Figure 1). There are advantages and limitations for each synthetic method, and the choice of method is selected based on the downstream applications. 

Due to their small size and improved cell-penetrating features, NPs are extremely useful in various biomedical applications including sensing [33,34], imaging [33,35], diagnostics [36,37], drug/compound delivery system [38,39], bioconjugation [40,41], hyperthermia [42,43], and biological therapies [33,36] (Figure 1). In the past few years, NPs have been widely used to improve sensing and imaging techniques mainly due to their remarkable localization capability [33]. Some other additional advantages using NPs include flexibility in surface modification of NP [44], easy control of size [45], and production of highly degradable NPs in vivo [46]. Moreover, NPs are widely used in bio-conjugation and combination with drugs and compounds as well as in facilitating their delivery to target [47]. In a review paper authored by Werengowska-Ciećwier et al., bioconjugation of various drugs and NPs as well as their detailed chemistry have been described [47]. The same review also discussed the application of NPs in drug delivery system for targeted therapies. The potential use of NPs in hyperthermia mainly in cancer cell killing have been extensively explored [48,49]. The use of magnetic hyperthermia is one of the hot approaches [48,50]. Using magnetic NPs, heat generation can be controlled and specifically target and kill cancer cells while limiting damage to the surrounding normal tissue [50,51]. As chemo- and radiotherapies are standard cancer treatments, it is anticipated that the use of NPs in combination with current treatments could enhance the treatment outcome while reducing side effects of chemo-and radio-therapies [51]. Similarly, NPs have also been shown to be effective against other infectious diseases such as *Pseudomonas aeruginosa* [52] and *Escherichia coli* [53] infections in addition to cancers. This highlights the important role of NPs in medical and biomedical application in short future. 

Generally, synthesis of NPs by biological routes has several advantages over both physical and chemical methods. First, the process is relatively simple, easy to scale up, efficient, and it consumes lesser energy **[26]**. Second, green technology is environmentally friendly as it uses lesser toxic chemicals and generating safer products and byproducts [54]. The green method is suitable and applicable for production of food, pharmaceuticals, and cosmetics [55]. Comparatively, the green method produces NPs that are generally less toxic, the end products being more suitable for a wide range of biomedical applications [26,56]. Chemical and physical methods can be very costly and usually involve the use of toxic and hazardous chemicals which tend to be more toxic to human cells. Additionally, the green method does not strictly require high temperature, pressure, and energy [57]. However, several parameters such as pH, chemical concentration, reaction time and reaction temperature are critical to consistently produce biologically functional NPs [58,59,60,61]. Furthermore, unlike using microbial system, generation of NPs from plants do not have to maintain microbial culture hence reducing the costs for microorganism isolation and culture media preparation [54]. The NPs generated from plants generally have size ranging from 1 to 100 nm (Table 1). There are also relatively large NPs that have sizes ranging from 100 to 500 nm [2,62,63]. NPs generated by all type of methods result in impurities that mainly cause toxicity to the human cells. These impurities can be removed by dialysis and filtering. The plant-derived NPs are less toxic, as shown from toxicity testing across several mammalian cell lines such as NIH3T3 [4], HEK293 [3], and primary cells such as peripheral blood mononuclear cells (PBMCs) [63] and rat aortic vascular smooth muscle cells (VSMCs) [4]. 

There is an extensive list of NPs possessing various biological actions as mentioned above. However, the focus of this review emphasizes the NPs’ antibacterial activities. This short review provides updates on the recent NPs with promising antibacterial activity synthesized by green technology using whole plant or other extracts of plants such as leaves [5,63], fruits [64,65], roots [66,67], barks [62,68], seeds [69,70], rhizomes [71,72], peels [73,74], flowers [75], and callus [76,77]. Here, we also discuss the challenges of adopting NPs for clinical applications. 

## 2. Bactericidal Properties and Synergistic Enhancement of Common Antibiotics

NPs derived from plants that show promising antibacterial activities have high potential to be developed into future antibacterials mainly due to their low toxic effects [143]. NPs have been previously reported to inhibit gram-positive bacteria such as *Staphylococcus* spp. [4,5], *Streptococcus* spp. [113,124], and *Bacillus* spp. [114,120], and gram-negative bacteria such as *Escherichia* spp. [97,122], *Pseudomonas* spp. [6,68], *Salmonella* spp. [68,104], *Shigella* spp. [97,129], *Proteus* spp. [75,136], and *Vibrio* spp. [101,129]. More promisingly, NPs have also been shown to inhibit antibiotic-resistant bacteria such as Methicillin-resistant *S. aureus* (MRSA) [81,130] and drug-resistant *E. coli* [123]. Table 1 summaries the antibacterial action of NPs reported in PubMed-indexed journals in the past two years (2016–2017). A total of 107 articles was obtained from Pub-Med search engine through National Center for Biotechnology Information (NCBI) website using four keywords (nanoparticles, green synthesis, plant, and antibacterial) [144]. Out of the 107, 17 articles have been excluded as they do not contain relevant information for this review. These articles included retracted papers, review papers, and other non-plant-derived NP research papers. The remaining 90 articles are reviewed, and the information is tabulated in Table 1 and categorized based on the type of NPs. Since there are various synthetic methods to generate NPs as mentioned in Section 1, the protocol of constructing the NPs tabulated in Table 1 is different from one to another even though they are derived from the same part of extracts. Overall, the most reported NPs are Ag-NPs, Au-NPs, followed by other metal/metal oxide-based NPs and nanocomposites. Most of the NPs have size of less than 100 nm except for a few NPs as shown in Table 1 [2,62,63,81]. From Table 1, it is also shown that most of the NPs were produced from the plant’s leaves rather than other parts of the plants, regardless of type of NPs. 

Table 2 shows both gram-positive and gram-negative bacterial species that have been targeted by various NPs, and the frequency of bacterial species being studied is tabulated. Gram-negative species that have been targeted the most are *E. coli* followed by *P. aeruginosa*, whereas gram-positive species that are mostly targeted are *S. aureus* followed by *B. subtilis* and *B. cereus*. Comparatively, the type and total number of gram-negative bacterial species that are being targeted are more than gram-positive species. This highlights the potential of NPs as antibacterial agents since they could effectively permeate and kill gram-negative bacterial species which are notorious of their difficult-to-penetrate multilayer membranes [145]. This notion can be further supported by recent finding by Acharya et al. which showed a potent killing of AgNPs towards *K. pneumonia* [146]. FE-SEM analysis demonstrated that the NPs overlaid with *K. pneumonia* with damaged cell surfaces and disrupted cells due to the interaction with NPs. Similarly, it has also been shown by SEM that AgNPs damaged *E. coli* by causing a large leakage on cell membrane and the bacteria were disorganized to several parts [147]. The potent antibacterial killings of NPs demonstrate their potential to be developed into antibacterials against gut-related bacteria (e.g., *E. coli*, *P. aeruginosa*, *B. cereus*, *K. pneumoniae*, *S. flexneri*, and *S. typhi*) and skin infection–related bacteria (*S. aureus*). The collective findings from Table 2 also show that Ag-NPs are the most bactericidal NPs against the gut bacteria such as *E. coli*, *P. aeruginosa*, *B. cereus*, *K. pneumoniae*, *S. flexneri*, *S. pyogenes*, *S. typhi*) and skin-related bacteria such as *S. aureus* and *S. epidermidis*. Followed by Ag-NPs, Au-NPs were reported to be effective in killing *E. coli* and *S. aureus*. Interestingly, Table 2 shows that some specific types of NPs were more effective against a particular bacterial type compared to others. For example, ZnO-NPs was effective against *P. aeruginosa* while Pd-NPs was effective against *S. aureus*.

In addition to exhibiting antibacterial effects, NPs derived from plants can also serve as carriers to deliver antibacterial molecules or drugs to the target cells either via conjugation or nanoemulsions, hence synergistically enhance the antibacterial effect [97,139]. For example, Kalita and coworkers demonstrated that a gold NP was able to enhance the bacterial killing effects of Amoxicillin against both gram-positive (*Staphylococcus* spp. and *Bacillus* spp.) and gram-negative bacteria (*E. coli*) [139]. Patra and colleagues reported the potential of silver NP synthesized from corn leaves of *Zea mays* in foodborne pathogenic bacterial killings when used in combination with Kanamycin and Rifampicin [97]. More interestingly, the NP was able to reverse the development of antibiotics resistance by killing MRSA clinical isolates *in vitro* and *in vivo* using murine MRSA infection models [148]. The exact mechanism of NPs’ synergism with antibiotics remains exploratory. It could be due to (a) generation of additional bactericidal Ag+ ions by NPs [149], (b) generation of bactericidal hydroxyl radicals by NPs [150], and (c) effective blocking of the efflux pump for drug-resistant bacterial killing [143]. This synergistic effect could ultimately help in reducing the dosage of antibacterials that may potentially toxic to host system. 

The exact mechanisms of NPs against various bacteria remain unknown. There have been several studies supporting the possible mechanisms of bactericidal effects including (a) attachment of large number of NPs on bacterial surface that interrupts respiration and other permeability-dependent functions [97], (b) generation of electrostatic attraction between negatively charged bacterial cells and the positively charged NPs [151], (c) inactivation and degradation of bacterial essential proteins [152], and (d) breakage or damage of bacterial genes following the efficient penetration of NPs [153]. For example, it has been shown that silver NPs permeated into bacterial cells and resulted in significant DNA damages by interacting with sulphur- and phosphorus-containing compounds [154,155]. Similarly, it has also been shown that silver NPs released highly reactive Ag+ ions and radicals for the antibacterial effects [156]. These ions have also been reported to interact with sulphur-containing proteins in the bacterial cell wall that caused multiple functionality impairs [152]. Raffi and colleagues also showed that the silver NPs could inactivate the bacterial enzymes and generate toxic hydrogen peroxide leading to bacterial cell death [157].

## 3. Plant-derived Nanoparticles as Future Antibacterials

To date, numerous NPs have been approved by either Food and Drug Administration (FDA) in United States, or European Medicines Agency (EMA) in the European Union for various clinical applications including imaging (e.g., Resovist), iron-replacement therapy for anaemia treatment (e.g., Vifor), delivery of anticancer drugs (e.g., Onivyde and MEPACT) [158,159], vaccines for viral diseases (e.g., Epaval against hepatitis A and Inflexal V against influenza) [160,161], fungal infection (e.g., AmBisome) [162], and so on. However, none of the currently approved NPs are used for controlling bacterial infection [163]. The only liposomal NP formulations that is undergoing clinical trial is CAL02, which has been designed for bacterial pneumonia management [163]. In this section, we discuss the potential challenges of developing NPs into clinically approved antimicrobial agents. Table 3 summaries some of the limitations and challenges of using plant-derived NPs for antimicrobials development. Undoubtedly, the nano-scale size of NPs has facilitated the cell-penetrating capacity including crossing blood-brain barrier (BBB) [164,165], hence improving the target specificity and biological activity. However, the small size may be one of the challenges in the clinical trials. It has been reported that NPs have poor stability and bioavailability under physiological conditions when they are administered into host system [166]. Studies have shown that NPs have been targeted and degraded by various enzymes and proteins from human blood before reaching to target sites [167,168]. These significantly reduced the biological functions of NPs. To overcome this limitation, several modification methods have been adopted such as conjugation with stabilizer (e.g., serum albumin) [169,170], synthesis of stable nanoemulsions [169,170], modification of surface chemistry and functionalization [171], and development of composite NPs [172]. In addition to size, it has also been reported that the NPs’ shape and morphology could determine their biological actions and toxicity profile [172,173,174]. Some NPs have high tendency to form aggregates as a result of the particle surface chemistry. This may cause unwanted toxicity and drastically limit the access of NPs into the target cells [175]. Toxicity is also attributed to several characteristics of NPs (e.g., chemistry, retention rate, biodistribution, stability, and specificity), mode of administration, and target sites [176]. 

There are numerous limitations for NPs productions for biomedical application (summarized in Table 3). As the biological functions of NPs highly depend on their shape, size, permeability, and physicochemical properties, manufacturing NPs in industrial scale must strictly adhere to the tight-controlled, consistent, and reproducible standard operating procedure (SOP) and Good Manufacturing Practice (GMP). Another challenge is the heterogeneity of diseases in human. The clinical effect of NPs on infected humans could be very complicated as different individuals present varied profiles (mainly due to the individual’s immunity) even though they have been infected from the same source. This will require detailed and organized planning to validate the clinical value of NPs. Following the increase of potential NPs, the experimental design that requires high throughput setup for biological screening is also increasingly demanding. This is also closely linked with automation that allows cost-effective NPs production and, computing and modelling technology that would predict NPs’ efficacies or toxicities on target cells. The improvement in high throughput screening and computation will surely boost the development of nanotechnology in biomedical applications. 

From an industrial point of view, the chosen synthesis method of NPs-based antibacterials must be compatible for large-scale production. As most NPs possess complex chemical make-up and arrangement of components, retaining these key characteristics in the process of scaling up remains a huge challenge in the manufacturing field. Secondly, the production method has to consistently produce high quality (e.g., size, uniformity, and physicochemical properties) of NPs. The formulation process must be recorded meticulously to ensure high-level of reproducibility. The advancement of modern technology contributed from large high-tech companies and academia will continuously support the production of high-quality NPs in a consistent and timely fashion. 

## 4. Conclusions and Future Perspectives

This mini-review provides a recent update on the NPs derived from plants that possess promising antibacterial action. Antibacterial NPs produced from green technology have great potential to be developed into future antibacterials and are able to synergistically enhance the efficacy of antibiotics. While the exact mechanisms remain unknown, a great effort is currently underway to produce a highly potent and robust NPs for clinical use. Understanding the bactericidal mechanism of NPs is also important to control and overcome the emerging issue of bacterial resistance to NPs [177]. As highlighted above, the limitations and potential challenges need to be overcome to maximize the use of NPs to clinical applications. Uniformity, stability, specificity, and toxicity of NPs are the main biological properties in deciding the fate of NPs in clinical application. From an industrial perspective, the development of sustainable process and modern instrumentation is crucial to produce a practical amount of NPs for clinical use [178]. The advancement of nanotechnology and biotechnology are anticipated to boost the use of NPs in biomedical applications in the future.

## Figures and Tables

**Table 1:** Antibacterial effects of nanoparticles synthesized by green method using plants against various bacteria reported in PubMed-indexed publications from 2016 to 2017.

Nanoparticles	Size (nm)	Source	Scientific Name	Common Name	Target Bacteria	References
Ag-NPs	26–28	Leaves	*Coleus aromaticus*	Cuban oregano	*Escherichia coli* (*E. coli*), *Staphylococcus aureus* (*S. aureus*)	[78]
	12.46	Leaves	*Salvinia molesta*	Kariba weed	*E. coli*, *S. aureus*	[79]
	70.7–192.02	Leaves	*Aloe vera*	Aloe	*Pseudomonas aeruginosa* (*P. aeruginosa*), *Streptococcus epidermidis* (*S. epidermidis*)	[63]
	5–50	Leaves	*Mentha pulegium*	Pennyroyal	*E. coli*, *S. aureus*, *Streptococcus pyogenes* (*S. pyogenes*)	[5]
	5–40	Leaves	*Cucurbita pepo*	Summer squash	*E. coli*, *S. aureus*, *Bacillus cereus* (*B. cereus*), *Listeria monocytogenes* (*L. monocytogenes*), *Salmonella typhi* (*S. typhi*), *Salmonella enterica* (*S. enterica*)	[80]
	112.6	Crude	*Ammania baccifera*	Monarch redstem	*S. aureus*, *P. aeruginosa*, MRSA	[81]
	10–70	Oil cake	*Cocos nucifera*	Coconut	*Aeromonas* sp., *Acinetobacter* sp., *Citrobacter* sp.	[82]
	3.2–16	Seeds	*Pimpinella anisum*	Aniseed	*K. pneumonia*, *P. aeruginosa*, *S. typhi* *Streptococcus pyogenes* (*S. pyogenes*), *Acinetobacter baumannii* (*A. baumannii*)	[69]
	2–25	Crude	*Matricaria camomilia*	Camomile	*E. coli*, *S. aureus*, *Bacillus subtilis* (*B. subtilis*), *P. aeruginosa*	[83]
	50	Crude	*Salvadora persica* L.	Toothbrush tree	*E. coli*, *S. aureus*	[84]
	25	Rhizomes	*Zingiber officinale*	Ginger	*E. coli*, *S. aureus*, *K. pneumonia*	[71]
	20	Leaves	*Gloriosa superba*	Flame lily	*E. coli*, *B. subtilis*	[85]
	5–25	Leaves	*Parkia roxburghii*	Tree bean	*E. coli*, *S. aureus*	[86]
	10–20	Tubers	*Dioscorea alata*	Yams	*E. coli*, *Staphylococcus auricularis* (*S. auricularis*)	[87]
	35–42.5	Powder	*Theobroma cacao*	Cacao	*E. coli*, *S. aureus*, *Staphylococcus epidermidis* (*S. epidermidis*), *P. aeruginosa*	[88]
	10–50	Leaves	*Adathoda vasica Linn*	Vasaka	*Vibrio parahaemolyticus* (*V. parahaemolyticus*)	[89]
	14.63	Crude	*Eleutherococcus senticosus*	Siberian ginseng	*E. coli*, *S. aureus*, *V. parahaemolyticus* *Bacillus anthracis* (*B. anthracis*)	[90]
	20–30	Seeds	*Coffea arabica*	Arabian coffee	*E. coli*, *S. aureus*	[70]
	20–100	Leaves	*Sonneratia apetala*	Sonneratia mangrove	*Shigella flexneri* (*S. flexneri*), *E. coli*, *S. aureus*, *Vibrio cholera* (*V. cholera*), *S. epidermidis*, *B. subtilis*	[62]
	50–400	Bark	*Heritiera fomes*	Sundari	*E. coli*, *S. aureus*, *V. cholera*, *S. epidermidis*, *B. subtilis*	[62]
	3–6	Crude	*Allium sativum* L.	Garlic	*E. coli*, *E. faecalis*, *Bacillus cereus* (*B. cereus*), *S. flexneri*	[91]
	3–22	Crude	*Zingiber officinale* Rosc.	Ginger	*E. coli*, *E. faecalis*, *B. cereus*, *S. flexneri*	[91]
	3–18	Crude	*Capsicum frutescens* L.	Cayenne pepper	*E. coli*, *E. faecalis*, *B. cereus*, *S. flexneri*	[91]
	10–20	Roots	*Salvadora persica* L.	Toothbrush tree	*E. coli*, *S. aureus*, *P. aeruginosa*, *Micrococcus luteus* (*M. luteus*)	[92]
	5–30	Crude	*Rumex dentatus*	Toothed dock	*P. aeruginosa*, *Bacillus thuringiensis* (*B. thuringiensis*)	[93]
	49	Flowers	*Millettia pinnata*	Karanja	*E. coli*, *P. aeruginosa*, *Proteus vulgaris* (*P. vulgaris*), *S. aureus*, *K. pneumonia*	[75]
	16.4	Seeds	*Pongamia pinnata*	Seashore Mempari	*E. coli*	[94]
	5–60	Rhizomes	*Dryopteris crassirhizoma*	Japanese fern	*P. aeruginosa*, *B. cereus*	[72]
	1–69	Leaves	*Ficus religiosa*	Peepul tree	*E. coli*, *B. subtilis*, *S. typhi*, *Pseudomonas fluorescens* (*P. fluorescens*)	[95]
	12–38	Powder	*Styrax benzoin*	Benzoin gum	*E. coli*, *P. aeruginosa*, *S. aureus*	[96]
	6.4–27.2	Callus	*Taxus yunnanensis*	Himalayan yew	*E. coli*, *S. aureus*, *S. paratyphi*, *B. subtilis*	[76]
	10–20	Ginseng berry	*Panax ginseng*	Meyer berries	*E. coli*, *S. aureus*	[4]
	45.26	Corn leaves	*Zea mays* L.	Maize	*E. coli*, *S. aureus*, *S. typhimurium*, *L. monocytogenes*, *B. cereus*	[97]
	20–80	Shoot tip	*Caesalpinia mimosoides* Lam.	Mimosa thorn	*E. coli*, *L. monocytogenes*	[98]
	37	Leaves	*Coriandrum sativum*	Coriander	*Propionibacterium acnes* (*P. acnes*)	[99]
	22.89	Aerial parts	*Artemisia tournefortiana*	-	*E. coli*, *B. subtilis*, *S. pyogenes*, *P. aeruginosa*	[100]
	20	Leaves	*Derris trifoliata*	Common derris	*E. coli*, *S. aureus*, *S. enterica*, *Vibrio parahaemolyticus* (*V. parahaemolyticus*)	[101]
	121	Roots	*Rheum palmatum*	Chinese Rhubarb	*S. aureus*, *P. aeruginosa*	[66]
	12.46	Leaves	*Salvinia molesta*	Giant salvinia	*E. coli*, *S. aureus*	[102]
	32.5	Roots	*Decalepis hamiltonii*	Indian Sarsaparilla	*E. coli*, *S. aureus*, *P. aeruginosa*, *B. cereus*, *B. licheniformis*	[67]
	16	Crude	*Heterotheca inuloides*	Mexican arnica	*E. coli*, *S. aureus*	[103]
	10–30	Fruit juices	*Vitis vinifera* and *Solanum lycopersicum*	Grape and tomato	*Pseudomonas septica* (*P. septica*), *S. aureus*, *M. luteus*, *Enterobacter aerogenes* (*E. aerogenes*), *B. subtilis*, *S. typhi*	[104]
	2.1–45.2	Callus	*Artemisia annua*	Sweet wormwood	*Arthrobacter arilaitensis* (*A. arilaitensis*), *Staphylococcus equorum* (*S. equorum*), *Microbacterium oxydans* (*M. oxydans*)	[77]
	15.2	Bark	*Crataeva nurvala*	Ayurveda	*P. aeruginosa*	[105]
	6–8	Fruit	*Tamarindus indica*	Tamarind	*P. aeruginosa*, *S. aureus*, *M. luteus*, *Enterobacter aerogenes* (*E. aerogenes*) *B. subtilis*, *B. cereus*, *S. typhi*	[106]
	12–80	Callus	*Nicotiana tabacum*	Tobacco	*E. coli*, *Agrobacterium rhizogenes* (*A. rhizogenes*)	[107]
	410–450	Leaves	*Lantana camara*	Verbanaceae	*E. coli*, *S. aureus*, *P. aeruginosa*	[2]
	25–40	Crude	*Actinidia deliciosa*	Kiwi fruit	*P. aeruginosa*	[6]
	15–28	Stem bark	*Ficus krishnae*	Krishna fig	*E. coli*, *S. aureus*, *S. typhimurium*	[68]
	2–15	Callus	*Catharanthhus roseus*	Madagascar periwinkle	*E. coli*	[108]
	28	Leaves	*Convolvulus arvensis*	Field bindweed	*E. coli*	[109]
	25	Leaves	*Artemisia vulgaris*	Common wormwood	*E. coli*, *S. aureus*, *P. aeruginosa*, *K. pneumonia*, *Haemophilus influenza* (*H. influenza*)	[110]
	20	Leaves	*Costus afer*	-	*E. coli*, *S. aureus*, *P. aeruginosa*, *K. pneumonia*, *B. subtilis*	[111]
	23–42	Leaves	*Exocoecaria agallocha*	Blinding tree	*P. aeruginosa*, *S. aureus*, *S typhi*, *B. cereus*	[112]
	10–80	Aerial parts	*Anthemis atropatana*	-	*E. coli*, *S. aureus*, *P. aeruginosa*, *S. pyogenes*	[113]
	40–60	Leaves	*Arbutus unedo*	Strawberry tree	*E. coli*, *S. epidermis*, *B. subtilis*, *P. aeruginosa*	[114]
	88.8	Leaves	*Cicer arietinum*	Chickpea	*E. coli*, *P. aeruginosa*	[115]
	5–30	Leaves	*Taraxacum officinale*	Dandelion	*Xanthomonas axonopodis* (*X. axonopodis*), *Pseudomonas syringae* (*P. syringae*)	[116]
	20–44.49	Leaves	*Prosopis cinerraria*	Khejri tree	*E. coli*, *K. pneumonia*, *S. epidermidis*	[117]
	15–25	Leaves	*Croton bonplandianum*	Bantulasi	*E. coli*, *S. aureus*	[118]
Au-NPs	5–10	Ginseng berry	*Panax ginseng*	Meyer berries	*E. coli*, *S. aureus*	[4]
	5–25	Leaves	*Parkia roxburghii*	Tree bean	*E. coli*, *S. aureus*	[71]
	10–75	Leaves	*Ginkgo biloba Linn*	Ginkgo tree	*Brevibacterium linens* (*B. linens*)	[119]
	3–37	Leaves	*Nigella arvensis*	Love-in-a-mist	*E. coli*, *S. aureus*, *P. aeruginosa*, *Serratia marcescens* (*S. marcescens*), *B. subtilis*, *S. epidermidis*	[120]
	25	Fruit	*Dimocarpus longan*	Longan	*S. aureus*, *B. subtilis*, *E. coli*	[64]
	5–25	Leaves	*Cerasus serrulata*	Japanese cherry	*E. coli*, *S. aureus*	[121]
	7–20	Crude	*Actinidia deliciosa*	Kiwi fruit	*P. aeruginosa*	[6]
	8–25	Peel	*Citrus maxima*	Pomelo	*E. coli*, *S. aureus*	[122]
	20–30	Crude	*Coptis chinensis*	Gold thread	Drug-resistant *E. coli*	[123]
Ag_2_O-NPs	42.7	Roots	*Ficus benghalensis*	Banyan	*Streptococcus mutans* (*S. mutans*), *Lactobacilli* sp.	[124]
NiO-NPs	9.69	Crude	*Moringa oleifera*	Drumstick tree	*S. aureus*, *S. pneumonia*, *Escherichia hermannii* (*E. hermannii*), *E. coli*	[125]
	10–20	Leaves	*Eucalyptus globulus*	Blue glum	*E. coli*, *S. aureus*, MRSA, *P. aeruginosa*	[126]
ZnO-NPs	20.06	Leaves	*Prunus x yedoensis Matsumura*	Yoshino cherry	*B. linens*, *S. epidermidis*	[127]
	400–500	Leaves	*Sonneratia apetala*	Sonneratia mangrove	*S. flexneri*	[62]
	47.27	Leaves	*Laurus nobilis*	Bay tree	*S. aureus*, *P. aeruginosa*	[128]
	50	Fruit	*Rosa canina*	Dog rose	*E. coli*, *L. monocytogenes*, *P. aeruginosa*	[65]
	*-*	Leaves	*Lobelia leschenaultiana*	Lobelia	*P. aeruginosa*, *Shigella sonnei* (*S. sonnei*), *P. vulgaris*, *V. parahaemolyticus*	[129]
	27–85	Fruit, seed, and pulp	*Citrullus colocynthis* L.	Schrad	MRSA, *P. aeruginosa*, *E. coli*, *B. subtilis*	[130]
Cu-NPs	21–30	Leaves	*Terminalia catappa*	Tropical almond	*E. coli*	[131]
	18.9–32.09	Leaves	*Prosopis cineraria*	Khejri tree	*E. coli*, *K. pneumonia*, *S. epidermidis*	[117]
CuO-NPs	30 –222.5	Leaves	*Seidlitzia rosmarinus*	Keliab	*E. coli*, *S. aureus*	[132]
Pt-NPs	2–7	Crude	*Taraxacum laevigatum*	Red-seeded dandelion	*P. aeruginosa*, *B. subtilis*	[133]
FeO-NPs	*-*	Peel	*Punica granatum*	Pomegranate	*P. aeruginosa*	[134]
Pd-NPs	5	Leaves	*Sapium sebiferum*	Chinese tallow tree	*S. aureus*, *P. aeruginosa* *Bacillus subtilis*	[135]
	30	Seeds	*Phyllanthus emblica*	Indian Gooseberry	*S. aureus*, *P. aeruginosa*, *B subtilis* *Proteus mirabilis*	[136]
	27	Peel	*Moringa oleifera*	Horseradish tree	*E. coli*, *S. aureus*	[73]
CeO_2_-NPs	45	Peel	*Moringa oleifera*	Horseradish tree	*E. coli*, *S. aureus*	[74]
	24	Leaves	*Olea europaea*	Olive	*E. coli*, *S. aureus*, *K. pneumonia*, *P. aeruginosa*	[137]
Ce_2_O_3_-NPs	8.6–10.5	Crude	*Euphorbia amygdaloides*	Wood spurge	*Pediococcus acidilactici* (*P. acidilactici*)	[138]
Pectin/Ag-NPs	20–80	Shoot tip	*Caesalpinia mimosoides* Lam.	Mimosa thorn	*E. coli*, *L. monocytogenes*	[98]
Ag/Ag_2_O-NPs	8.2–20.5	Leaves	*Eupatorium odoratum*	Christmas bush	*E. coli*, *S. typhi*, *S. aureus*, *B. subtilis*	[139]
Ag/Au-NPs	10	Leaves	*Gloriosa superba*	Flame lily	*E. coli*, *B. subtilis*	[85]
Chitosan/Ag-NPs	378–402	Crude	*Rumex dentatus*	Toothed dock	*P. aeruginosa*, *B. thuringiensis*	[140]
Chitosan/CeO_2_-NPs	3.61–24.4	Leaves	*Sida acuta*	Common wireweed	*E. coli*, *B. subtilis*	[141]
PCL/Cur/GLE-Ag-NPs	200	Leaves	*Vitis vinifera*	Grape	*E. coli*, *S. aureus*, *P. aeruginosa*, *B. subtilis*, *S. enterica*	[93]
GLE-Ag-NPs	30	Leaves	*Vitis vinifera*	Grape	*E. coli*, *S. aureus*, *P. aeruginosa*, *B. subtilis*, *S. enterica*	[93]
Cellulose/Cu-NPs	20–40	Leaves	*Terminalia catappa*	Tropical almond	*E. coli*	[131]
Ag-MnO_2_-NPs	5–40	Leaves	*Cucurbita pepo*	Summer squash	*E. coli*, *S. aureus*, *B. cereus*, *L. monocytogenes*, *S. typhi*, *S. enterica*	[142]

**Table 2:** Gram-negative and gram-positive bacterial species targeted by NPs synthesized from plants via green technology.

**Gram-Negative Species**	**Ag**	**Au**	**Cu**	**Pt**	**Pd**	**Ag_2_O**	**NiO**	**ZnO**	**CuO**	**FeO**	**CeO_2_**	**Ce_2_O_3_**
*E. coli*	46	6	2	-	1	-	2	2	1	-	2	-
Drug-resistant *E. coli*	-	1	-	-	-	-	-	-	-	-	-	-
*E. hermannii*	-	-	-	-	-	-	1	-	-	-	-	-
*P. fluorescens*	1	-	-	-	-	-	-	-	-	-	-	-
*P. aeruginosa*	23	2	-	1	2	-	1	4	-	1	1	-
*P. syringae*	1	-	-	-	-	-	-	-	-	-	-	-
*P. septica*	1	-	-	-	-	-	-	-	-	-	-	-
*K. pneumoniae*	6	-	1	-	-	-	-	-	-	-	1	-
*P. vulgaris*	1	-	-	-	-	-	-	1	-	-	-	-
*P. mirabilis*	-	-	-	-	1	-	-	-	-	-	-	-
*S. flexneri*	4	-	-	-	-	-	-	1	-	-	-	-
*S. sonnei*	-	-	-	-	-	-	-	1	-	-	-	-
*S. paratyphi*	1	-	-	-	-	-	-	-	-	-	-	-
*S. typhi*	6	-	-	-	-	-	-	-	-	-	-	-
*S. typhimurium*	2	-	-	-	-	-	-	-	-	-	-	-
*S. enterica*	2	-	-	-	-	-	-	-	-	-	-	-
*V. parahaemolyticus*	3	-	-	-	-	-	-	1	-	-	-	-
*V. cholera*	2	-	-	-	-	-	-	-	-	-	-	-
*Aeromonas* sp.	1	-	-	-	-	-	-	-	-	-	-	-
*Acinetobacter* sp.	1	-	-	-	-	-	-	-	-	-	-	-
*A. baumannii*	1	-	-	-	-	-	-	-	-	-	-	-
*Citrobacter* sp.	1	-	-	-	-	-	-	-	-	-	-	-
*E. aerogenes*	2	-	-	-	-	-	-	-	-	-	-	-
*A. rhizogenes*	1	-	-	-	-	-	-	-	-	-	-	-
*H. influenza*	1	-	-	-	-	-	-	-	-	-	-	-
*X. axonopodis*	1	-	-	-	-	-	-	-	-	-	-	-
*S. marcescens*	-	1	-	-	-	-	-	-	-	-	-	-
*Lactobacilli* sp.	-	-	-	-	-	1	-	-	-	-	-	-
**Gram-Positive Species**	**Ag**	**Au**	**Cu**	**Pt**	**Pd**	**Ag_2_O**	**NiO**	**ZnO**	**CuO**	**FeO**	**CeO_2_**	**Ce_2_O_3_**
*S. aureus*	35	6	-	-	3	-	2	1	1	-	2	-
*S. epidermidis*	5	1	1	-	-	-	-	1	-	-	-	-
MRSA	1	-	-	-	-	-	1	1	-	-	-	-
*S. pyogenes*	4	-	-	-	-	-	-	-	-	-	-	-
*S. mutans*	-	-	-	-	-	1	-	-	-	-	-	-
*S. pneumonia*	-	-	-	-	-	-	1	-	-	-	-	-
*B. thuringiensis*	1	-	-	-	-	-	-	-	-	-	-	-
*B. cereus*	9	-	-	-	-	-	-	-	-	-	-	-
*B. subtilis*	11	2	-	1	2	-	-	1	-	-	-	-
*B. licheniformis*	1	-	-	-	-	-	-	-	-	-	-	-
*B. anthracis*	1	-	-	-	-	-	-	-	-	-	-	-
*E. faecalis*	3	-	-	-	-	-	-	-	-	-	-	-
*L. monocytogenes*	3	-	-	-	-	-	-	1	-	-	-	-
*M. luteus*	3	-	-	-	-	-	-	-	-	-	-	-
*P. acnes*	1	-	-	-	-	-	-	-	-	-	-	-
*A. arilaitensis*	1	-	-	-	-	-	-	-	-	-	-	-
*M. oxydans*	1	-	-	-	-	-	-	-	-	-	-	-
*B. linens*	-	1	-	-	-	-	-	1	-	-	-	-
*P. acidilactici*	-	-	-	-	-	-	-	-	-	-	-	1

**Table 3:** Challenges of developing nanoparticles into clinically used antibacterial agents.

**Structural Challenge**	
Size	Smaller size enhances the cell penetration, but may have decreased stability or bioavailability
Shape	Certain shape of NPs may improve the functionality due to total surface exposure area
Aggregate	NPs that form aggregate increase the overall particle size, hence limiting the cell permeation and may increase toxicity
**Biological Challenge**	
Biodistribution	Poor dispersion due to limited entry (e.g., skin barrier)
Bioavailability	Poor bioavailability results in rapid loss of function
Specificity	High specificity results in less off-target effects and more effective
Clearance	High retention rate ensures the high efficiency
Toxicity	Accumulation of toxic materials may damage the host
**Technological Challenge**	
Heterogeneity of human disease	Variation within disease may complicate treatment
Scale-up	Optimization of NPs synthesis and production with uniform size without aggregates in controlled and consistent fashion
Throughput	Synthesis of NP is multistep and laborious which does not allow high-throughput optimization
Prediction	Prediction using computer modelling on NP efficiency is extremely challenging
**Industrial Challenge**	
Quantity	Large scale production may result in inconsistent size and physicochemical properties of NPs
Processes	Reproducible and consistent manufacturing processes requires modern technology and instrumentation
Quality	Continuous production of high level uniformity and functionality of NPs
